# Myoepithelioma Arising in the Buccal Mucosa: A Case Report and Review of the Literature

**DOI:** 10.7759/cureus.73263

**Published:** 2024-11-08

**Authors:** Shunsuke Ochiai, Manabu Yamada, Kenichiro Suga, Masafumi Nishikawa, Seiji Asoda

**Affiliations:** 1 Division of Dentistry and Oral Surgery, Shizuoka Cancer Center, Shizuoka, JPN; 2 Department of Dentistry and Oral Surgery, National Hospital Organization, Tochigi Medical Center, Tochigi, JPN; 3 Department of Pathology, National Hospital Organization, Tochigi Medical Center, Tochigi, JPN; 4 Department of Dentistry and Oral Surgery, Keio University School of Medicine, Tokyo, JPN

**Keywords:** buccal, buccal mucosa, myoepithelioma, pleomorphic adenoma, salivary gland tumour

## Abstract

Although myoepithelioma is defined as a benign tumour made up primarily of neoplastic myoepithelial cells, its actual histopathological characteristics are highly diverse. It can be considered a rare disease. The most common site of occurrence is the parotid gland, followed by the minor salivary glands of the palate. Very little has been reported about its occurrence in the buccal mucosa. We present a case of a 55-year-old woman with myoepithelioma arising in the buccal mucosa who was referred to our hospital for swelling of the buccal mucosa. Contrast-enhanced computed tomography revealed a mass measuring approximately 18 mm in diameter in the left lateral buccal mucosa, and the interior of the mass showed slight heterogeneous enhancement. Magnetic resonance imaging revealed an almost low signal intensity comparable to that of the muscle on T1-weighted imaging and an irregular mixture of low and high signal intensities on T2-weighted imaging. The tumour was diagnosed as a pleomorphic adenoma based on biopsy results and resected under general anaesthesia. The final diagnosis was myoepithelioma, which requires careful differentiation from pleomorphic adenoma. At 32 months after surgery, no evidence of recurrence was detected.

## Introduction

Myoepitheliomas are benign tumours composed mostly of neoplastic myoepithelial cells [1]. Myoepitheliomas account for only approximately 1.5% of all salivary gland tumours [2]. Myoepitheliomas that occur in the buccal mucosa are extremely rare. We present a case of myoepithelioma arising in the buccal mucosa and review the relevant literature on this rare disease.

## Case presentation

**Figure 1:**
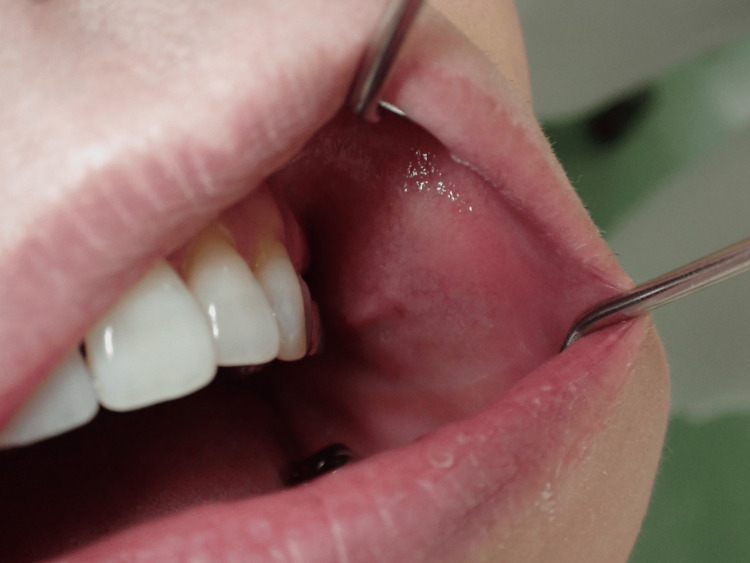
Intraoral photograph taken at the initial examination. An elastic soft mass was found in the left buccal mucosa.

A 55-year-old woman presented to our department with an enlargement of the left buccal mucosa. For about seven months, she had been aware of a gradual increase in swelling of the left buccal mucosa, but it was left untreated because it was painless. There was no swelling in the left side of the cheek, no left-right difference in facial appearance, and no facial nerve palsy. An elastic soft mass was found in the left buccal mucosa, which was mobile to bimanual palpation, nontender, and covered with a normal mucosa measuring 30 mm in the greatest dimension. There was no abnormality in the salivary discharge from the parotid papillae at palpation (Figure 1).

**Figure 2:**
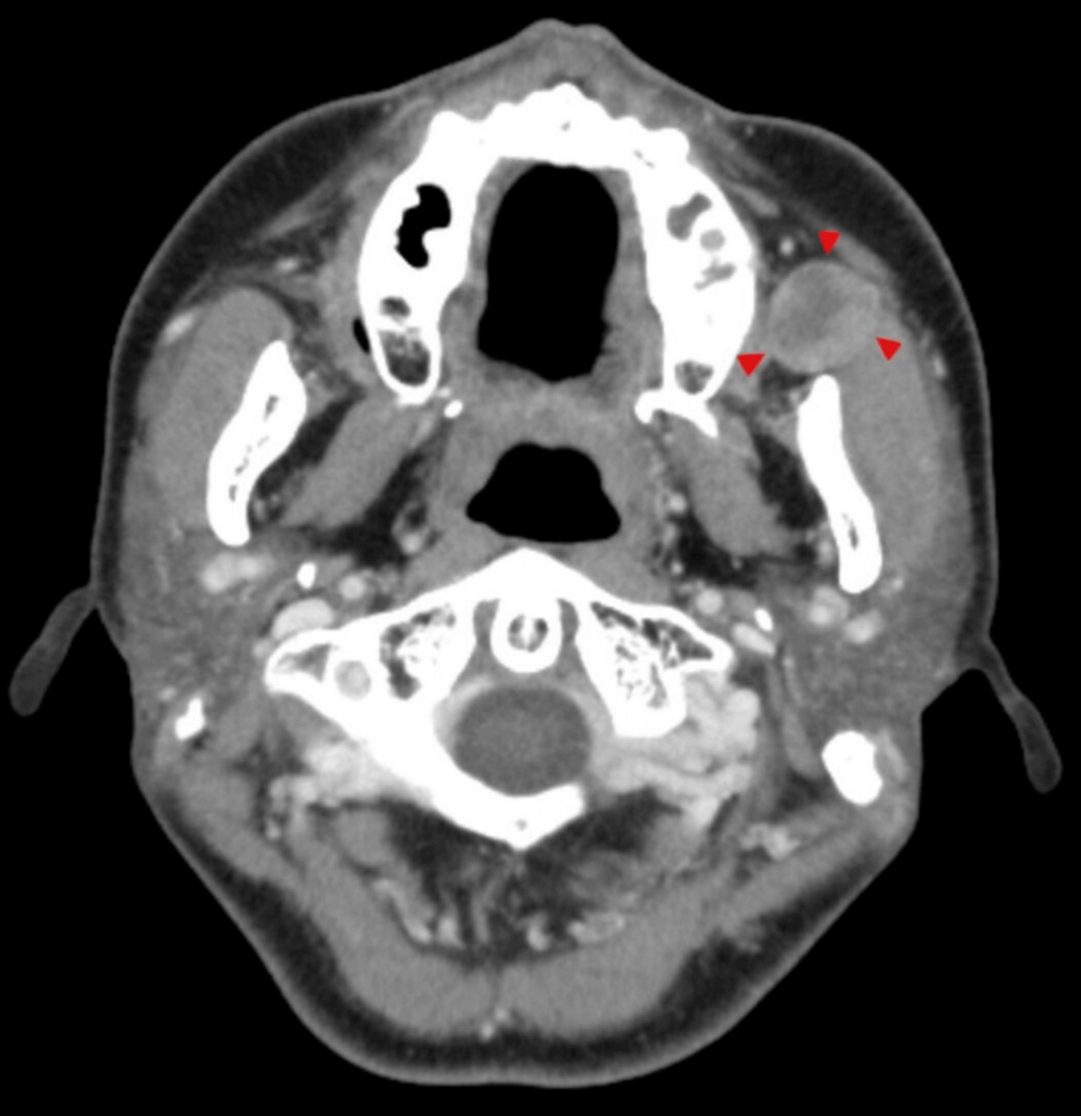
CT findings. A solid, well-circumscribed mass measuring approximately 18 mm in diameter was observed in the left buccal space (arrowhead).

No significant enlargement of the bilateral cervical lymph nodes or other lesions was observed in the head and neck region. Contrast-enhanced computed tomography (CT) revealed a solid, well-circumscribed mass measuring approximately 18 mm in diameter in the left buccal space, and the inside of the mass showed slight heterogeneous enhancement (Figure 2). No abnormalities were observed in the cervical lymph nodes.

**Figure 3:**
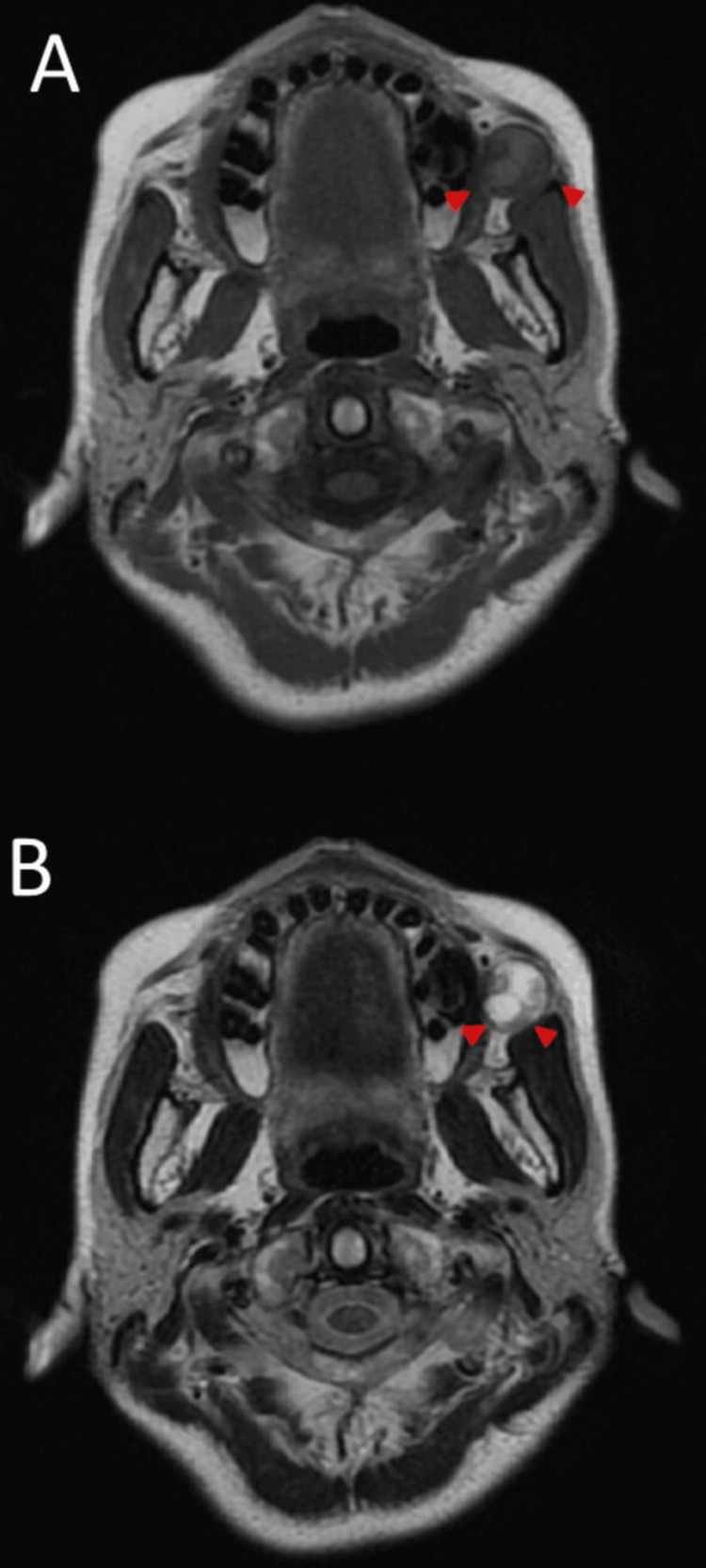
MRI findings. Most of the tumour shows a low signal intensity comparable to muscle on T1-weighted imaging (arrowhead) (A). An irregular mixture of low and high signal intensities is observed on T2-weighted imaging (arrowhead) (B).

On magnetic resonance imaging (MRI), most of the tumours showed a low signal intensity similar to that of the muscle on T1-weighted imaging and an irregular mixture of low and high signal intensities on T2-weighted imaging (Figure 3).

**Figure 4:**
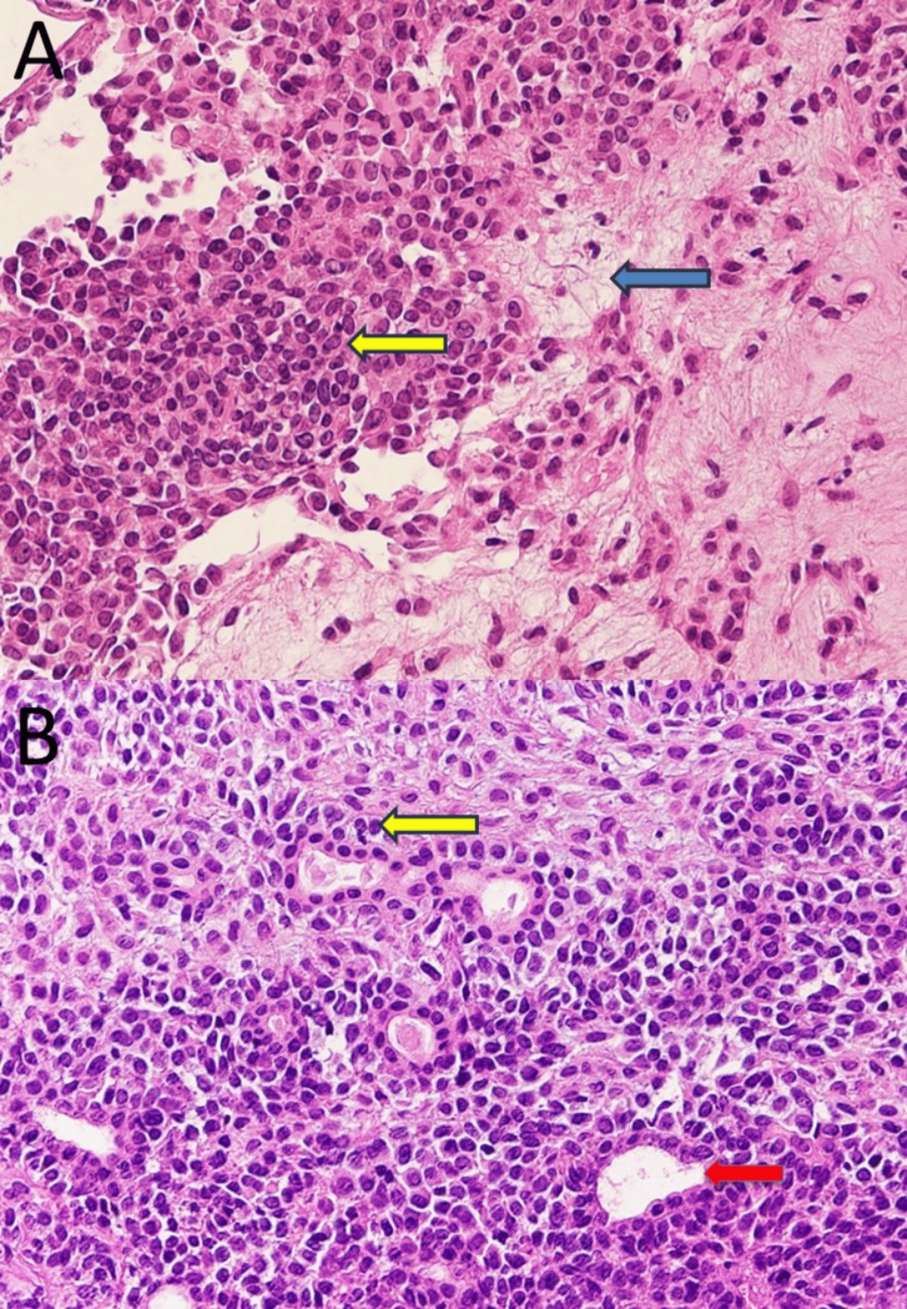
Histopathological findings (hematoxylin and eosin stain) at biopsy. The proliferation of myoepithelial cells (yellow arrow) and mild myxomatous changes (blue arrow) were observed (A, ×40). Glandular duct structures (red arrow) were observed (B, ×40).

Laboratory findings did not reveal abnormalities. Based on the above findings, the clinical diagnosis was a buccal mucosa tumour. An incisional biopsy was performed under local anaesthesia, which confirmed the diagnosis of pleomorphic adenoma (Figure 4).

**Figure 5:**
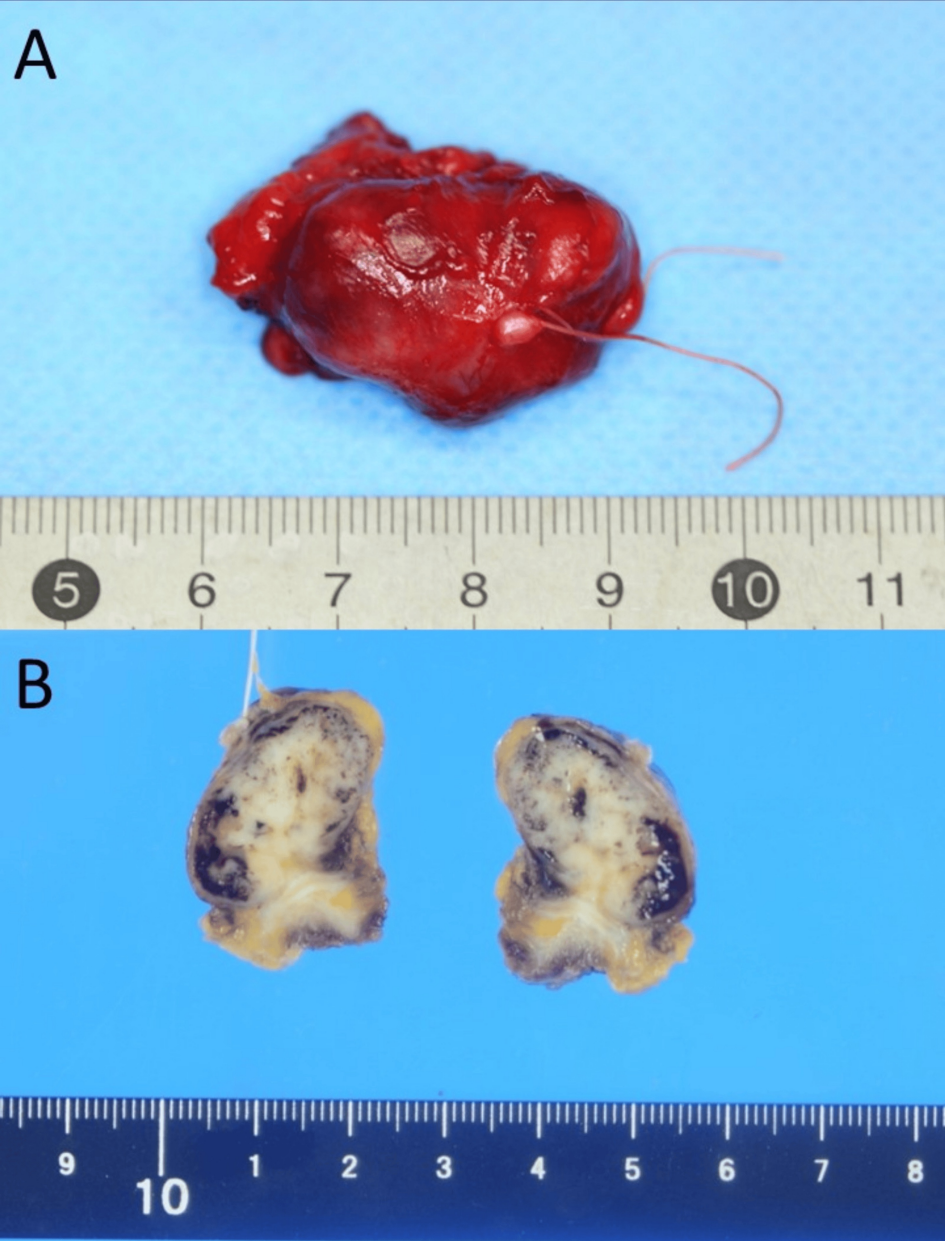
The resected specimen. The tumour was a well-circumscribed, capsulated mass with a smooth surface (A). The cut surface appeared solid and whitish to greyish-yellowish-white in colour (B).

Two months after the initial diagnosis, the buccal mucosa tumour was resected under general anaesthesia. The incision line was set in a spindle shape to include the incision line at the time of biopsy. The tumour extended to the anterior margin of the masseter muscle and was resected as a single mass, together with the surrounding tissues and capsule. The defect mucosa was slight, and a primary suture was performed. At the time of resection, the parotid duct was detected running from the depth of the masseter muscle to the inside of the tumour, so it was also resected and the orifice of the parotid conduit was transplanted. The resected specimen was 30 × 20 × 20 mm in size. The tumour was a well-circumscribed, capsulated mass with a smooth surface. The cut surface appeared solid and whitish to greyish-yellowish-white in colour (Figure 5).

**Figure 6:**
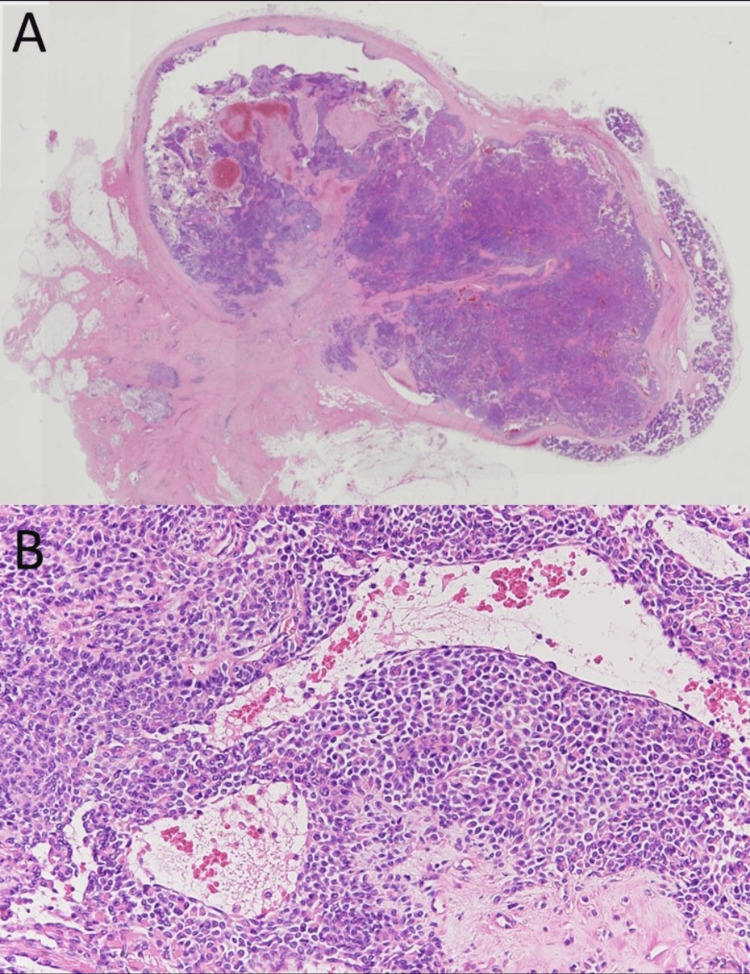
Histopathological findings (hematoxylin and eosin stain). The tumour was covered by a fibrous capsule with well-defined borders (A, ×4). The proliferation of myoepithelial cells was observed (B, ×40).

Histopathologically, it was a well-defined tumour covered by a fibrous capsule, with mainly myoepithelial cell proliferation accompanied by mild myxomatous changes in some areas (Figure 6). The tumour cells had round or spindle-shaped nuclei, arranged in bundles. Although glandular duct structures were observed in some tissues, in general, few such structures were found and they were inconspicuous. The tumour cells were a mixture of plasmacytoid cells with vitreous-like sporangia and ubiquitous round nuclei, and spindle-shaped cells with spindle nuclei arranged in bundles. No dissemination of the tumour cells into the surrounding tissues was observed.

**Figure 7:**
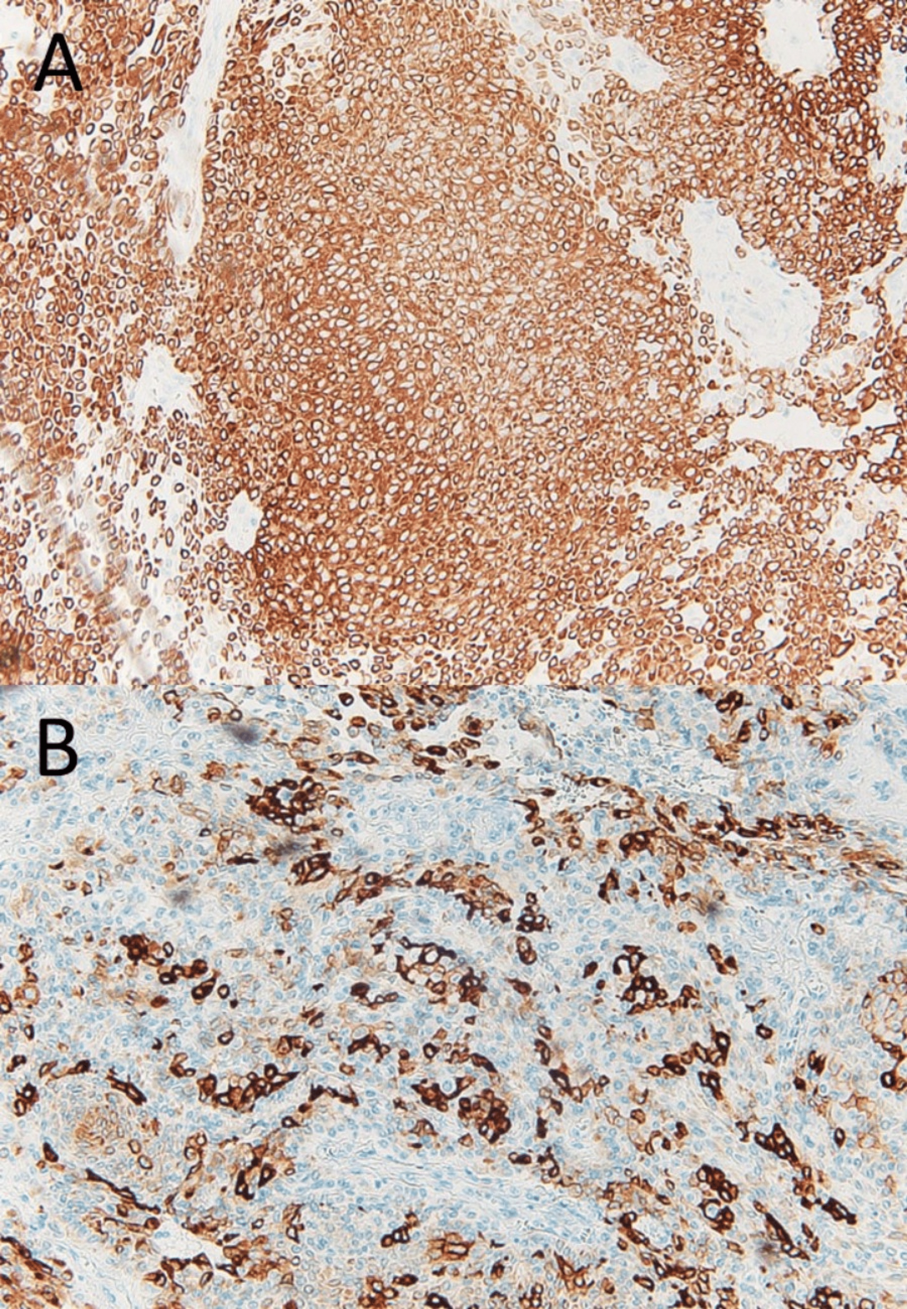
Histopathological findings by immunohistochemical staining. The tumour cells were positive for cytokeratin 5 and 6 (A, ×40) and AE1/AE3 (B, ×40).

**Figure 8:**
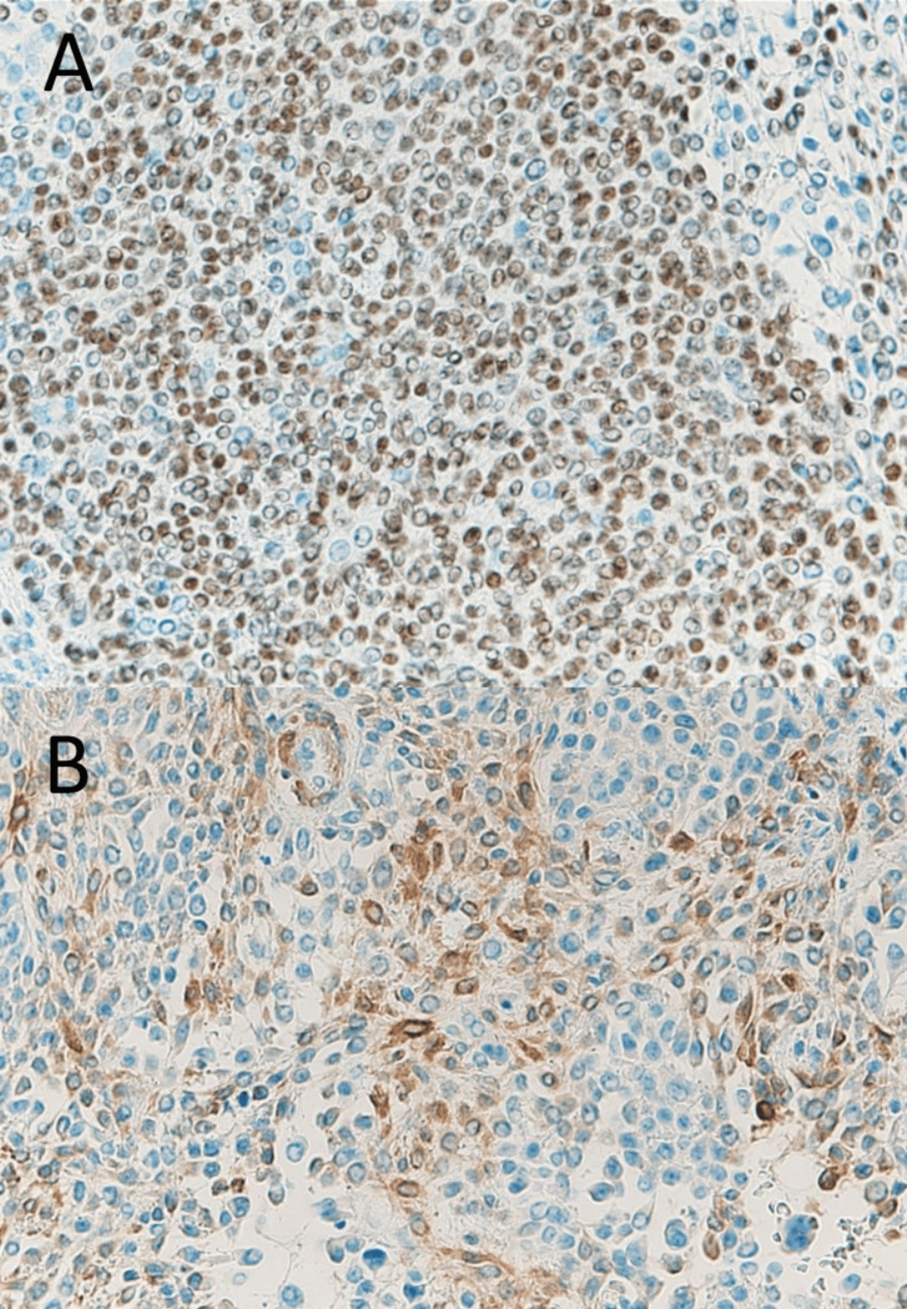
Histopathological findings by immunohistochemical staining. The tumour cells were positive for p63 (A, ×40) and calponin (B, ×40).

Immunohistochemical staining revealed that almost the entire tumour was positive for cytokeratin 5 and 6, AE1/AE3 (pan-keratin staining) (Figure 7), and p63, with focal positivity for calponin (Figure 8). Based on these findings, the final diagnosis was myoepithelioma. No evidence of recurrence was identified for 32 months after surgery.

## Discussion

According to the 2017 WHO classification, among benign epithelial tumours, myoepithelioma is classified as an independent tumour along with pleomorphic adenoma [1]. It is defined as a benign tumour mainly composed of neoplastic myoepithelial cells and accounts for approximately 1.5% of all salivary gland tumours [2]. The parotid gland is the most frequent site of occurrence, accounting for 40% of cases, followed by the palate [3]. Myoepithelioma occurring in the cheek region is rare. To our knowledge, only 11 cases, including the present case, have been reported in the relevant literature (Table 1) [4-12]. Among these, lesions in the buccal mucosa within the buccal muscle are extremely rare, with only four cases reported in the relevant literature [5,6,12], including the present case.

**Table 1:** Reports of myoepithelioma in the cheek region.

Study	Report year	Age	Sex	Occurrence site	Symptoms	Sickness period	Morphologic pattern	Treatment	Recurrence
Tajima et al. [4]	1984	56	Female	Buccal mucosa	Unknown	Unknown	Mixed spindle, plasmacytoid	Unknown	Unknown
Sugiura et al. [5]	2000	54	Female	Buccal mucosa	Painless	5 years	Mixed spindle, plasmacytoid	Resection	No recurrence
Kawashima et al. [6]	2002	34	Female	Cheek	Occasional pain	4 years	Spindle	Resection	No recurrence
Isogai et al. [7]	2004	47	Female	Cheek	Unknown	1 year	Plasmacytoid	Resection	No recurrence
Ferri et al. [8]	2006	81	Female	Cheek	Painless	2 years	Spindle	Resection	No recurrence
Sun et al. [9]	2009	41	Male	Cheek	Unknown	Unknown	Unknown	Resection	No recurrence
	2009	22	Female	Cheek	Unknown	Unknown	Unknown	Resection	No recurrence
Park et al. [10]	2011	23	Male	Buccal mucosa	Painless	Several years	Plasmacytoid	Resection	No recurrence
Wakoh et al. [11]	2014	58	Male	Cheek	Painless	3 months	Plasmacytoid	Resection	No recurrence
Iguchi et al. [12]	2014	31	Female	Cheek	Painless	5 years	Spindle	Resection	No recurrence
Present case	2023	55	Female	Buccal mucosa	Painless	7 months	Mixed spindle, plasmacytoid	Resection	No recurrence

In general, the age of onset of myoepithelioma ranges from nine to 85 years, and both sexes are equally affected [13]. They are clinically characterised by slow development and are often asymptomatic [13]. The 11 cases of the cheek region myoepithelioma ranged in age from 22 to 81 years (median = 45.6), which is similar to the trends in past reports on general myoepithelioma. However, there was some bias against sex, with eight of the 11 patients being women, a trend different from previous reports.

Histopathological characteristics of myoepithelioma on hematoxylin and eosin staining are as follows: the tumour is covered with a thin fibrous capsule, as well as being spindle-shaped, plasmacytoid, vitelliform, epithelioid, clear cell, or a mixture of these [14,15]. In general, many of these are considered to be composed of one cell type. Of the 11 cases of cheek region myoepithelioma, six were composed of one cell type: three were spindle-shaped and three were plasmacytoid. Mixed spindle-shaped and plasmacytoid cell types were found in three cases, including the present case. On immunohistochemical staining, myoepithelial cells should be positive for α-smooth muscle actin (SMA), muscle-specific actin (MSA, HHF35), calponin, cytokeratin 5, 14, and 17, p63, S-100 protein, GFAP, and vimentin [16]. In the current case, immunohistochemical staining was positive for calponin, cytokeratin 5 and 6, p63, and AE1/AE3 (pan-keratin staining). In salivary gland tumours, the frequency of positivity and staining differs depending on the cell type of the tumour [17]. Therefore, the pathological diagnosis must always be confirmed based on the general histological picture using multiple immunohistochemical markers.

Given these findings, a malignant tumour was initially ruled out because the resected tumour was covered with a thin fibrous capsule that completely surrounded the periphery and there was no infiltrative proliferation, nuclear atypia, increased nuclear fission, or necrosis. Second, the predominant proliferation of myoepithelial cells ruled out a tumour without differentiation into myoepithelial cells, such as Warthin's tumour, oncocytoma, or cystadenoma. Basal cell adenomas and pleomorphic adenomas are the most common benign salivary gland tumours that differentiate into myoepithelial cells. Basal cell adenomas are mainly accompanied by foci of basal cell-like cells or solid growth and show a cord-like structure and fenestrated arrangement. Basal cell adenomas were excluded because they are usually not associated with the appearance of plasmacytoid cells. The distinction between myoepithelioma and pleomorphic adenoma is particularly difficult, as in this case, the diagnosis at biopsy and the final pathological diagnosis were not consistent. In general, myoepitheliomas are considered to have very few or no glandular ductal structures and very limited chondroid-like or myxoid-like changes in the cells. These are considered to be the points of discrimination between the two types of tumours. Although myoepithelioma was previously defined as a tumour without glandular differentiation in the 1991 WHO classification [2], in the 2005 edition, the formation of a small number of glandular ducts was also recognised as myoepithelioma [18] and is currently redefined as a benign tumour composed mostly of neoplastic myoepithelial cells. Furthermore, myoepitheliomas tend to have more uniform tumour cell proliferation than pleomorphic adenomas, and myxomatous stromal components with mixed tumour cells appear less frequently [19]. However, myoepithelioma is considered an extension of the various histological types exhibited in pleomorphic adenoma, and there is no clear distinction between myoepithelioma and pleomorphic adenoma. In the present case, the diagnosis at biopsy was pleomorphic adenoma because glandular duct structures and myxomatous changes were observed in the excised sections. However, when the tissue was re-examined after total resection, it was found that the glandular duct structures observed at biopsy were only slightly present in the whole. This suggested that they were pre-existing tissue components. Because the tumour was mainly composed of myoepithelial cells, the final histopathological diagnosis was myoepithelioma. Salivary gland tumours are histopathologically diverse and some may show very similar histology, even when the diagnoses are different [20]. This makes it difficult to distinguish whether the tissue component of the lesion is part of the tumour or existing normal tissue. It was confirmed that making a comprehensive diagnosis and assessing the whole histological composition is important when making a histopathological diagnosis, rather than confirming the diagnosis based on the presence of partially histological features.

Resection is the first-line treatment of choice. In 10 of the cases reviewed, the tumour was resected along with the surrounding tissues. Although some reports suggest that the rate of recurrence of myoepithelioma is lower than that of pleomorphic adenoma [16], the majority of reports suggest that the rate of recurrence and invasiveness is higher [1,2]. Therefore, resection of myoepithelioma in conjunction with surrounding healthy tissues is recommended. Myoepithelioma is a benign tumour; however, repeated recurrences can lead to malignant transformation. Among the cases that we reviewed, there were no cases of recurrence in the cheek region and most myoepitheliomas were resected with the surrounding tissues. Resection, including surrounding tissues, and careful long-term follow-up are vital for the treatment of myoepithelioma.

## Conclusions

Although myoepithelioma is a benign salivary gland tumour, it can be difficult to differentiate it from other salivary gland tumours, such as polymorphous adenomas, due to its highly variable histopathology. It is necessary to thoroughly determine the diagnosis, considering that the results may differ from those at the time of biopsy. In addition, many reports indicate a high recurrence rate and a high degree of invasiveness. Therefore, it is advisable to resect the tumour, including the surrounding tissue.
